# Refining DNA Barcoding Coupled High Resolution Melting for Discrimination of 12 Closely Related *Croton* Species

**DOI:** 10.1371/journal.pone.0138888

**Published:** 2015-09-25

**Authors:** Maslin Osathanunkul, Chatmongkon Suwannapoom, Sarawut Ounjai, Jantarika A. Rora, Panagiotis Madesis, Hugo de Boer

**Affiliations:** 1 Department of Biology, Faculty of Science, Chiang Mai University, Chiang Mai 50200, Thailand; 2 State Key Laboratory of Genetic Resources and Evolution and Yunnan Laboratory of Molecular Biology of Domestic Animals, Kunming Institute of Zoology, Chinese Academy of Sciences, Kunming 650223, China; 3 Science and Technology Research Institute, Chiang Mai University, Chiang Mai, 50200, Thailand; 4 Institute of Applied Biosciences, Centre for Research & Technology Hellas, Thessaloniki, Greece; 5 Department of Organismal Biology, Evolutionary Biology Centre, Uppsala University, Norbyvägen 18D, SE-75236 Uppsala, Sweden; 6 The Natural History Museum, University of Oslo, P.O. Box 1172, NO-0318 Oslo, Norway; University of Milano Bicocca, ITALY

## Abstract

DNA barcoding coupled high resolution melting (Bar-HRM) is an emerging method for species discrimination based on DNA dissociation kinetics. The aim of this work was to evaluate the suitability of different primer sets, derived from selected DNA regions, for Bar-HRM analysis of species in *Croton* (Euphorbiaceae), one of the largest genera of plants with over 1,200 species. Seven primer pairs were evaluated (*matK*, *rbcL*1, *rbcL*2, *rbcL*3, *rpoC*, *trnL* and ITS1) from four plastid regions, *matK*, *rbcL*, *rpoC*, and *trnL*, and the nuclear ribosomal marker ITS1. The primer pair derived from the ITS1 region was the single most effective region for the identification of the tested species, whereas the *rbcL*1 primer pair gave the lowest resolution. It was observed that the ITS1 barcode was the most useful DNA barcoding region overall for species discrimination out of all of the regions and primers assessed. Our Bar-HRM results here also provide further support for the hypothesis that both sequence and base composition affect DNA duplex stability.

## Introduction

### Classification of *Croton* and uses in ethnomedicine

*Croton* (Euphorbiaceae) is one of the largest genera of flowering plants, with between 1,200 and 1,300 species. It is widespread in tropical areas, with habits ranging from large woody trees through climbing lianas to simple and prostrate weeds [1,2]. In Southeast Asia and Thailand there are at least 80 species and 30 species of *Croton*, respectively [3]. The complex subgeneric taxonomy of *Croton* relies on a provisional revision of the sections of the genus from the early 1990s [1]. Several studies, based on both classical taxonomy and phylogenetic analyses, have demonstrated the complex taxonomy of the *Croton* genus (e.g. [1,4]). An example of this complexity is highlighted by *C*. *acutifolius* Esser in Thailand. This species is superficially similar to *C*. *robustus* Kurz, *C*. *laccifer* L. and *C*. *caudatus* Geiseler which can however be distinguished by their fruit size, and *C*. *argyratus* Blume which is characterized by silvery pubescent mature leaves [3]. Additionally, *C*. *griffithii* Hook.f. has been confused with *C*. *oblongus* Burm.f. by several authors, as the two species show similarity in their indumentum and floral characters [3].

Many species of *Croton* are used in traditional herbal medicine around the world. In Asia, and Thailand in particular, there has been a resurgence of interest in traditional medicine, aided by government programs to promote research into medicinal plants as a potential source of new remedies. Among the most popular remedies are those based on *Croton* species. Many *Croton* species from this region have been used in traditional medicine and have had their biological activities assessed. These include *Croton caudatus* Geiseler, *Croton crassifolius* Geiseler, *C*. *kongensis* Gagnep., *C*. *oblongifolius* Roxb., *C*. *sublyratus* Kurz., *C*. *tiglium* L., and *C*. *tonkinensis* Gagnep. [5–10]. At the same time, it is widely known from scientific and folk literature that many species of *Croton* are toxic and cause irritation [11], and therefor the correct identification of the species is important to avoid negative health effects.

In Thailand, *C*. *stellatopilosus* H.Ohba, known locally as ‘*Plao Noi*’, is a popular natural remedy used for stomach disorders. Commercial products containing ‘*Plao Noi*’ are sold as tea bags, capsules, and in powder form in herbal markets and are claimed to have anti-ulcer, anti-cancer, and anti-inflammatory effects. However, confusion has arisen because the same vernacular name is used for a number of species. At least six *Croton* species, *C*. *columnaris* Airy Shaw, *C*. *delpyi* Gagnep., *C*. *longissimus* Airy Shaw, *C*. *kongensis* Gagnep., *C*. *stellatopilosus* and *C*. *thorelii* Gagnep., share the same Thai common name ‘*Plao Noi*’ [3]. The different species vary in their secondary metabolite spectra, which results in variation in their potential efficacy and safety.

### Identification of plant products in trade through DNA barcoding

Medicinal plant products are commonly sold in processed or modified forms such as powders, dried material, tablets, capsules and tea bags, making it almost impossible to accurately identify the constituent species using morphology [12,13]. Incorrect identification of the constituent plants may lead to the inclusion of undesirable, unrelated species, with potential health risks to end users. Substitution of the product’s ingredients either intentionally or inadvertently can have negative effects on both consumers and producers. Herbal products are often perceived to be safe due to their natural origin. However, counterfeited, substituted and adulterated products can put consumers in danger [14–16]. There is an urgent need to find an approach that could help with the quality control of these herbal products in order to ensure consumer safety and which can also produce reliable species identifications even when morphology-based identification is impossible.

Molecular identification through DNA barcoding is a powerful method for the identification of plant species, including medicinal plants and products. Many studies have shown the potential for DNA barcoding to effectively distinguish among medicinal plants, as well as to identify constituent species in processed herbal medicines [12,17–20]. Several reviews have highlighted the increasing and diverse applications of medicinal plant barcoding [21–23]. However, DNA barcoding in plants does have limitations. These include the inability to amplify marker regions due to degraded DNA in some processed samples [24], limited binding site universality [12,25,26], low rates of marker discrimination [12,27], overlapping intraspecific and infraspecific genetic variation in some groups of plants [28], and low applicability of chloroplast markers for the identification of species of hybrid origin [28]. Another limitation of DNA barcoding is the associated costs, as it requires a molecular laboratory, costly equipment, chemicals and disposables, and DNA sequencing facilities. The lack of access to DNA sequencing facilities and the high costs of sequencing often impedes the wider implementation of DNA barcoding in developing countries [29].

### Bar-HRM for species discrimination

Developing and validating sequencing-free methods that are reliable, yet faster and more economical than DNA barcoding is challenging, but will be beneficial for the advancement of herbal product identification routines in developing countries. High resolution melting (HRM) is an emerging method for monitoring DNA dissociation (“melting”) kinetics, and is a powerful technique for the detection of point mutations, indels, and methylated DNA [30,31]. In addition to standard PCR equipment and reagents, HRM requires a generic DNA intercalation fluorescent dye. This dye is added to previously amplified PCR products and as the double-stranded DNA samples dissociate with increasing temperature the dye is progressively released and fluorescence diminishes. These denaturation thermodynamics are based on the binding affinities of individual nucleotide pairs, which vary due to indels, mutations and methylations. These differences are inferred by fluorescent measurements collected at standard temperature increments, which are plotted as a melting curve. The curve’s shape and peak are characteristic for each sample, allowing for comparison and discrimination among samples. By using HRM, single base changes between samples can be readily detected and identified [32,33].

The first study reporting the use of Bar-HRM to evaluate herbal medicine substitution came from a recent investigation of species substitution among three medicinal species of Acanthaceae [29]. Bar-HRM has also been used in a number of comparable applications [34], such as for authentication of an EU Protected Designation of Origin product made from *Lathyrus clymenum* [35], for the identification of olive oil and adulterants [36], for subspecies cultivar identification in eggplants [37], for the identification of closely related species of *Sideritis* and *Helleborus* [38,39], for species distinction in Mediterranean pines [40], for the detection of allergenic hazelnut contamination [41], and for the identification of processed bean crops [42–44].

### Research questions and hypothesis

The use of Bar-HRM has been reported for species identification and detection in food and herbal and agricultural products. However, all previous studies have looked at species discrimination in complexes of limited species diversity [29,35–37,40–42]. In this study we test the hypothesis that reduced melting discrimination resolution among twelve closely related species in the same genus could be overcome by marker optimization and combination of data from multiple Bar-HRM markers. Here, we use a dataset made up of twelve species in the genus *Croton* to answer the following research questions relevant to the model group: 1) Can Bar-HRM primers sets that enable universal amplification of selected plastid and nuclear markers be designed?; 2) Can these twelve related *Croton* species be discriminated by Bar-HRM?; 3) Which single Bar-HRM marker has the highest rate of successful discrimination?; 4) Which minimum number of combined Bar-HRM markers that together give the highest rate of successful identification?

## Methods

### DNA mining of barcode regions

Sequences of four selected plastid DNA regions (*matK*, *rbcL*, *rpoC* and *trnL*) and one nuclear regions (ITS1) of *Croton* species from the family Euphobiaceae were extracted from GenBank (at the end of November 2014) using the key phrases “the name of locus” and “the name of genus” in the annotations. Generally, sequences obtained from public databases, including GenBank, are of low quality with no known associated herbarium vouchers. For this reason, all of the sequences were subjected to critical evaluation and any low-quality sequences were removed. After processing, multiple sequence alignments were made from the selected sequences using MEGA6 [45] and variable characters were calculated in order to design primers to be used for high resolution melting (HRM) analyses. The species names and accession numbers of all analyzed sequences are listed in S1 Table.

### Plant material and DNA isolation

Dried plant tissues for DNA extraction were kindly provided by Queen Sirikit Botanic Garden (QSBG) (Table 1). The plant material was ground with liquid nitrogen, and 100 mg of fine powder was then used for DNA extraction with the Nucleospin Plant II kit (Macherey-Nagel, Germany) following the manufacturer’s instructions. DNA concentrations of all samples were adjusted to a final concentration of 20 ng/μL. The DNA was stored at −20°C for further use.

**Table 1:** Plants included in this study.

Species	Abbrev.	Sample No.	Thai Vernacular name	Voucher No.
*Croton cascarilloides* Raeusch.^1^	CAS	1	Plao ngoen; ‘เปล้าเงิน’	QBG38236
*Croton caudatus* Geiseler^2^	CAU1CAU2	23	Plao; ‘เปล้า’	QBG8395QBG3389
*Croton crassifolius* Geiseler	CR1CR2	45	Pang khi; ‘ปังคี’	QBG20547QBG10911
*Croton delpyi* Gagnep.^3^	CD	6	Plao noi; ‘เปล้าน้อย’	QBG13889
*Croton griffithii* Hook.f.^2^	CG	7	Plao; ‘เปล้า’	QBG32192
*Croton hutchinsonianus* Hosseus^4^	CH1CH2	89	Plao lueat; ‘เปล้าเลือด’	QBG21732QBG2327
*Croton kongensis* Gagnep.^1^ ^,^ ^3^	CK1CK2CK3	101112	Plao noi; ‘เปล้าน้อย’, Plao ngoen; ‘เปล้าเงิน’	QBG6677QBG3807QBG33093
*Croton poilanei* Gagnep.^4^ ^,^ ^5^	CP	13	Plao luang; ‘เปล้าหลวง’, Plao yai; ‘เปล้าใหญ่’, Plao lueat; ‘เปล้าเลือด’	QBG12427
*Croton robustus* Kurz^5^	CRB1CRB2	1415	Plao yai; ‘เปล้าใหญ่’	QBG8309QBG5954
*Croton persimilis* Müll.Arg.^5^ ^,^ *	CRX1CRX2	1617	Plao luang; ‘เปล้าหลวง’, Plao yai; ‘เปล้าใหญ่’	QBG984QBG5997
*Croton tiglium* L.	CB	19	Hat sakhuen; ‘หัสคืน’	QBG10124
*Croton thorelii* Gagnep.^3^	CT	20	Plao noi; ‘เปล้าน้อย’	QBG29049

^1^ share the vascular name ‘*Plao Ngoen*’

^2^ share the vascular name ‘*Plao*’

^3^ share the vascular name ‘*Plao Noi*’

^4^ share the vascular name ‘*Plao Lueat*’

^5^ share the vascular names ‘*Plao Luang*’ and ‘*Plao Yai*’

* Synonym *Croton roxburghii* N.P.Balakr.

### Real-time PCR amplification and high resolution melting (HRM) analysis

PCR amplification, DNA melting and fluorescence measurements were performed in a total reaction volume of 20 μL on an Eco Real-Time PCR system (Illumina, San Diego, USA) to determine the characteristic melting temperature (T_m_) for each sample that could be used to distinguish among the plants (species?) in genus *Croton*. Each reaction mixture contained 20 ng of genomic DNA, 10 μL of MeltDoctor HRM Master Mix (Applied Biosystems, California, USA) and 0.2 μL of 10 mM forward and reverse primers. The nucleotide compositions of the five candidate pairs of barcoding primers are shown in Table 2. The real-time PCR reaction conditions were; an initial denaturing step at 95°C for 5 min followed by 35 cycles of 95°C for 30 s, 57°C for 30 s and 72°C for 20 s. Subsequently, the PCR amplicons were denatured for HRM at 95°C for 15 s and then annealed at 50°C for 15 s to form random DNA duplexes. Melting curves were generated after the last extension step. The temperature was increased from 60 to 95°C at 0.1°C/s. The melting curves were analyzed with the EcoStudy Software v5.0.

**Table 2:** Oligonucleotide sequences of primers used for HRM analyses.

Primer HRM	5' → 3'	T_a_ (°C)	Expected size (bp)
HRM_rpoCF	CCSATTGTATGGGAAATACTT	57	170
HRM_rpoCR	CTTACAAACTAATGGATGTAA		
HRM_matKF	CTTCTTATTTACGATTAACATCTTCT	57	170
HRM_matKR	TTTCTTTGATATCGAACATAATG		
HRM_trnLF	TGGGCAATCCTGAGCCAAATC	57	120
HRM_trnLR	AACAGCTTCCATTGAGTCTCTGCACCT		
HRM_rbcL1F	GCAGCATTCCGAGTAACTCCTCA	57	100
HRM_rbcL1R	TCCACACAGTTGTCCATGTACC		
HRM_rbcL2F	GGTACATGGACAACTGTGTGGA	57	150
HRM_rbcL2R	ACAGAACCTTCTTCAAAAAGGTCTA		
HRM_rbcL3F	TAGACCTTTTTGAAGAAGGTTCTGT	57	145
HRM_rbcL3R	TGAGGCGGRCCTTGGAAAGTT		
HRM_ITS1FHRM_ITS1R	GGTGAACCTGCGGAAGGATCATTGCCGAGATATCCATTGCCGAGAGTC	57	300

## Results and Discussion

### Data mining

Sequences from all five selected barcode regions were extracted from GenBank for species in the genus *Croton*, and the variable characters and average %GC content were calculated for all samples using MEGA6. Sequence data were present for most markers, except for *rpo*C. In total, 62 sequences of *matK*, 148 of *rbcL*, 240 of *trnL* and 770 of ITS1 were retrieved, of which 19, 39, 136 and 293 sequences of each respective region were deemed useful for further analysis (Table 3). The *rpoC* data were excluded from this study due to the absence of a sufficient number of *rpoC* reference sequences for the target species (only 1). A single set of primers for each of *matK*, *trnL*, and ITS1, along with three sets of primers for *rbcL* (*rbcL*1-*rbcL*3), were evaluated. These various primer combinations yielded amplicons ranging from 100 to 320 bp (Table 3). Reed and Wittwer [31] found that amplicons suitable for HRM analysis should be 300 bp or less for optimal results. The *trnL* and ITS1 primer sets yielded amplicons of variable length, whereas the *rbcL* and *matK* primers set yielded amplicons of consistent sizes (Table 3).

**Table 3:** Characteristics of sequences from selected plastid regions (*matK*, *rbcL*, *rpoC* and *trnL*) and nuclear genome region (ITS1) retrieved from GenBank (NCBI) from species of *Croton*.

Markers	*mat*K	*rbc*L1	*rbc*L2	*rbc*L3	*trn*L	ITS1	*rpoC*
Search result from GenBank	62	148	148	148	240	770	0
Sequences included in dataset	19	39	39	39	136	293	-
Expected product size	125	100	149	145	101–115	290–320	-
Characters (bp)	125	100	149	145	124	353	-
Variable characters (%)	19 (15.2)	14 (14)	13 (8.72)	12 (8.27)	29 (23.38)	239 (67.7)	-
Conserved F primer sites/total (%)	8/26 (30.77)	20/22 (90.9)	22/22 (100)	24/25 (96)	20/21 (95.24)	23/24 (95.83)	-
Conserved R primer sites/total (%)	25/27 (92.59)	24/25 (96)	24/25 (96)	20/21 (95.24)	26/27 (96.29)	20/24 (83.33)	-
Average %GC content (SD)	32.26 (1.02)	53.54 (0.59)	45.1 (0.67)	43.56 (0.72)	35.74 (1.1)	52.17 (2.82)	-

**Fig 1:**
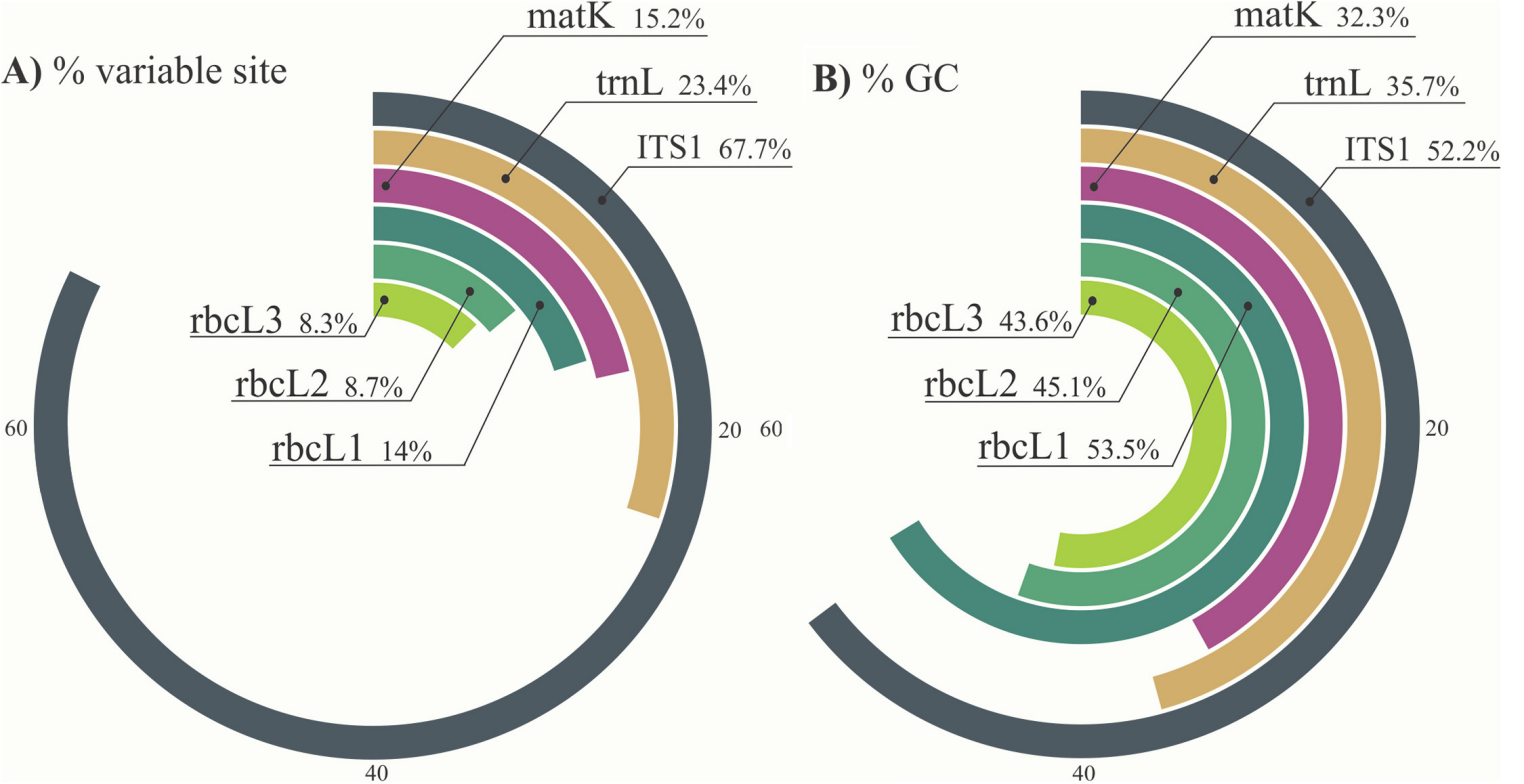
Comparison of variable characters (1A) and average %GC content (1B) of *Croton matK*, *rbcL1*, *rbcL2*, *trnL* and *ITS1* sequences retrieved from GenBank. The regions correspond to the same fragments that were amplified using the primers described in the present study.

Both the sequence length and the nucleotide variation within sequences influence the dissociation energy of the base pairs and result in different T_m_ values. Two hundred and thirty nine variable sites (67.7%) were observed within the analyzed fragment of the ITS1 region (353 characters) (Table 3). The ITS1 amplicon sequences were observed to have the highest nucleotide variation, and the relative nucleotide variation within amplicons was found to be: ITS1> *trnL* > *matK* > *rbcL*1 > *rbcL*2 > *rbcL*3 (Fig 1A).

The forward *matK* primer matched the consensus sequence of the target species at the binding sites in only 8 out of 26 sites (30.77%) (Table 3). High universality at the initial bases of the primer site is crucial for primer annealing and subsequent elongation initiation by the DNA polymerase. The *matK* locus is one of the most variable plastid coding regions and has high interspecific divergence and good discriminatory power. However, it can be difficult to amplify with the standard barcoding primers due to high substitution rates at the primer sites [46,47]. The average %GC content of amplicons was calculated in order to predict variation in melting curves for the different markers. The *matK* region had the lowest average %GC content, with 32.26%, followed by *trnL*, *rbcL*3, *rbcL*2, ITS1, and *rbcL*1 with 35.74, 43.56, 45.1, 52.17 and 53.54% respectively (Fig 1B).

### Evaluation of primers and amplicons for discrimination among four *Croton* species

**Fig 2:**
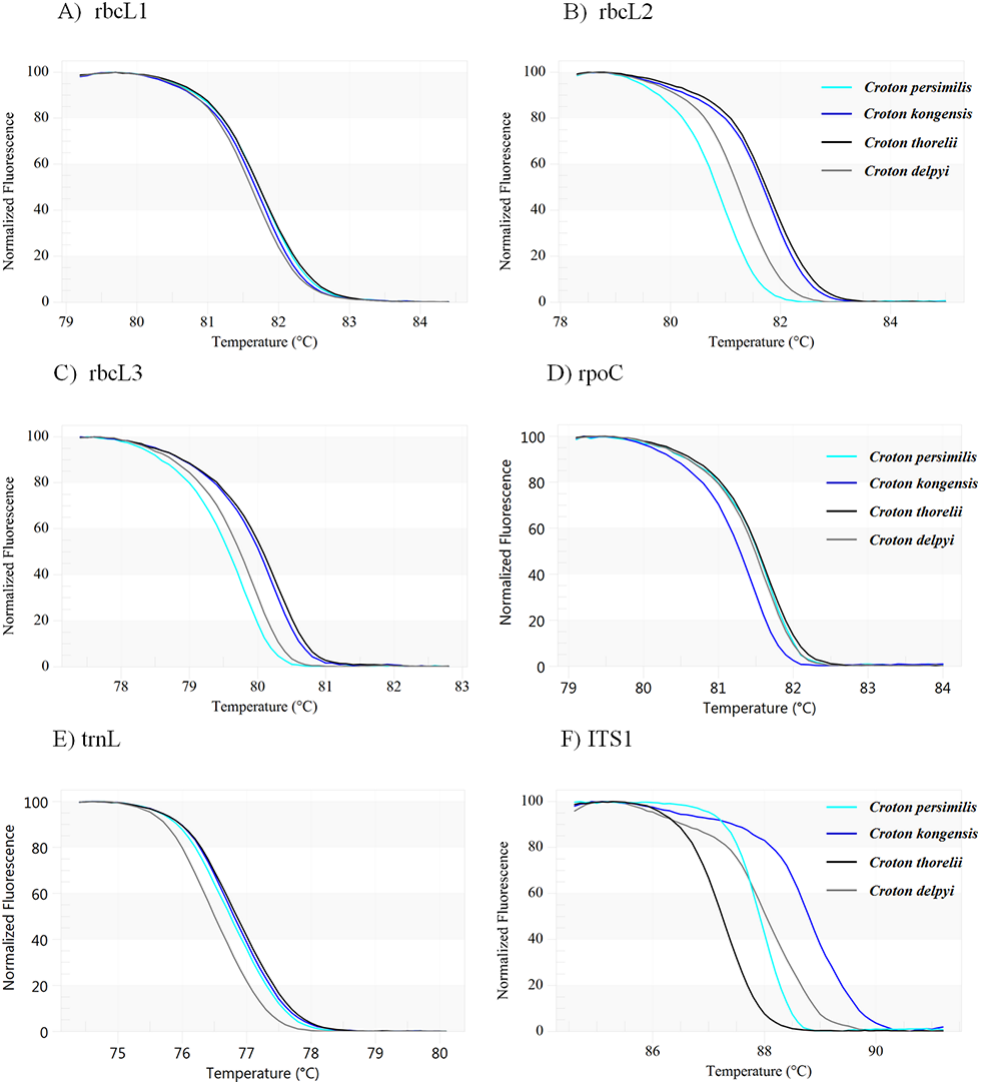
Melting curves of four *Croton* species generated by HRM using the following primer pairs: 2A) *rbcL*1; 2B) *rbcL*2; 2C) *rbcL*3; 2D) *rpoC*; 2E) *trnL* and 2F) ITS1.

**Fig 3:**
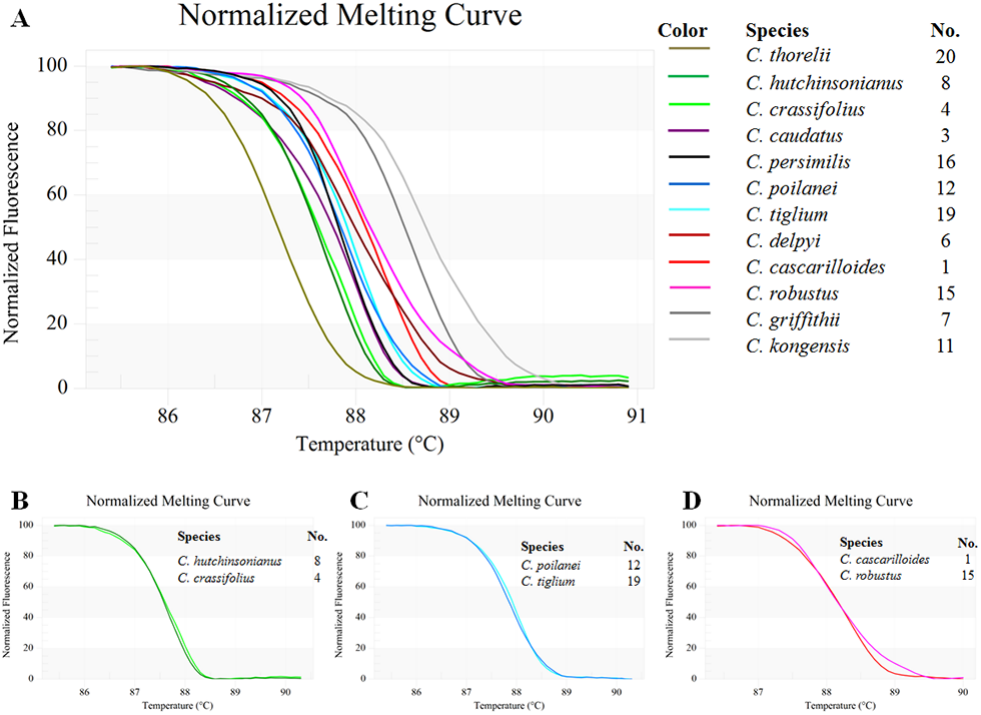
The normalized plot of each amplified fragment derived from ITS1 region shows the differentiation of melting temperature (T_m_) of each ITS1 amplicon from each species, generated by high resolution melting (HRM) analysis. **3A)** Twelve *Croton* species, **3B)** *C*. *hutchinsonianus* and *C*. *crassifolius*, **3C)** *C*. *poilanei* and *C*. *tiglium*, **3D)** *C*. *cascarilloides* and *C*. *robustus*.

**Fig 4:**
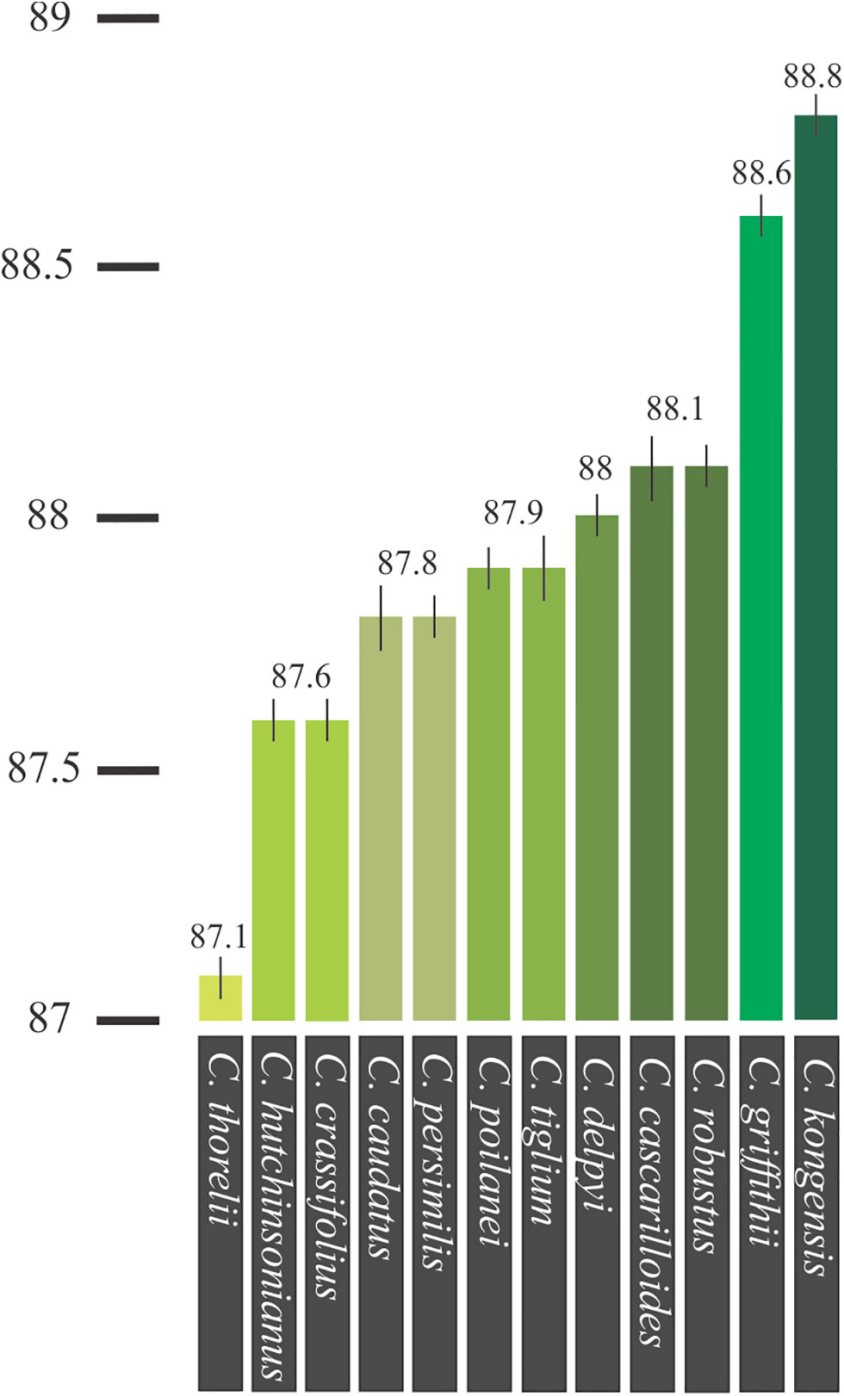
Average T_m_ of the ITS1 amplicon for all twelve *Croton* species.

The seven HRM primer sets were used for the amplification of DNA-fragments from four *Croton* species, *C*. *delpyi*, *C*. *kongensis*, *C*. *persimilis* and *C*. *thorelii*, and the resulting amplicons were analyzed using HRM to define the melting temperature (T_m_). These seven primer sets amplified fragments from *matK*, *rbcL*1, *rbcL*2, *rbcL*3, *rpoC*, *trnL* and ITS1 and yielded amplicons of 125 bp, 100 bp, 150 bp, 150 bp, 170 bp, 120 bp, and 300 bp, respectively. HRM analysis was performed in triplicate on each of the four taxa to establish the T_m_ for each primer set. The shapes of the melting curves were analyzed using EcoStudy Software v 5.0 to distinguish among the different plant species. The melting profiles of all amplicons are illustrated in Fig 2A–2F. The mean of the melting temperatures obtained from each primer pair, along with the melting curve shape, was used to measure the discriminatory power of each region regarding the species tested. The discrimination power of each region ranged from 0% (*rbcL*1) to 100% (ITS1). The primer pairs could be divided into four classes as follows: 1) none of the tested species could be distinguished from each other (*rbcL*1) (Fig 2A), 2) two out of the four species could be distinguished (*rpoC* and *trnL*) (Fig 2D and 2E), 3) three out of the four species could be distinguished (*rbcL*2 and *rbcL*3) (Fig 2B and 2C), and 4) all four species could be distinguished (ITS1) (Fig 2F). Although *matK* has been proposed as one of the best plant barcodes in terms of species discrimination [46,48], we found that the amplification of the fragment of *matK* that we targeted had such a low success rate that it could not be used for further analysis. Based on these results, it was predicted that the ITS1 primer pair would perform well in HRM analyses with an expanded sampling of *Croton* species. Several other studies have also shown the accuracy and universality of ITS1 for DNA barcoding in plants [12,17,19,48–51].

### Bar-HRM using ITS1 primers and an expanded *Croton* sampling

The ITS1 primer set yielded amplicons of the expected size, approximately 300 base-pairs long, and the amplicons were analyzed using HRM to determine the T_m_ of all 12 tested *Croton* species (Table 4). Fig 3 depicts the analysis by means of conventional derivative plots, which show the T_m_ value for the ITS1 fragment from each species. However, not all amplicons from the different species yielded distinctive HRM profiles. The melting curves were reproducibly achieved from each of the tested species in triplicate analyses.

**Table 4:** Average T_m_ with standard deviation (SD) of the ITS1 amplicon for all twelve *Croton* species.

Species	SampleNo.	T_m_ (°C)			SD	Average T_m_(°C)
		1	2	3		
*Croton cascarilloides*	1	88.1	88.2	88.1	0.06	88.1
*Croton caudatus*	3	87.8	87.8	87.7	0.06	87.8
*Croton crassifolius*	4	87.6	87.6	87.5	0.06	87.6
*Croton delpyi*	6	88	87.9	88.1	0.10	88.0
*Croton griffithii*	7	88.6	88.6	88.5	0.06	88.6
*Croton hutchinsonianus*	8	87.6	87.6	87.5	0.06	87.6
*Croton kongensis*	11	88.8	88.7	88.9	0.10	88.8
*Croton poilanei*	13	87.9	87.9	88	0.06	87.9
*Croton robustus*	14	88.1	88.2	88	0.10	88.1
*Croton persimilis*	16	87.7	87.8	87.8	0.06	87.8
*Croton tiglium*	19	87.8	87.8	87.9	0.06	87.8
*Croton thorelii*	20	87.1	87.1	87.2	0.06	87.1

The tested species formed nine clusters, denoted as A-I, when both T_m_ and the curve shape were taken into account. These were: A) *C*. *thorelii* (Sample No. 20, cf. Table 1), B) *C*. *hutchinsonianus* (8) and *C*. *crassifolius* (4) (Fig 3B), C) *C*. *caudatus* (3), D) *C*. *persimilis* (16), E) *C*. *poilanei* (12) and *C*. *tiglium* (19) (Fig 3C), F) *C*. *delpyi* (6), G) *C*. *cascarilloides* (1) and *C*. *robustus* (15) (Fig 3D), H) *C*. *griffithii* (7), and I) *C*. *kongensis* (11) (Fig 4).

To corroborate the results of the HRM analysis, the ITS1 sequences of the 12 tested species were used for further analysis. Sequence data from six species, *C*. *cascarilloides* (CAS), *C*. *caudatus* (CAU), *C*. *crassifolius* (CRA), *C*. *hutchinsonianus* (HUT), *C*. *kongensis* (KON) and *C*. *persimilis* (PER), were obtained. Pairwise comparison of the species’ respective ITS1 sequences showed the number of variable sites ranged from 4 sites for PER-HUT to 21 for CAS-CRA (Table 5). HRM can detect differences among samples of as little as one base pair, but in order to allow for intra-specific and geographical variations within species, an arbitrary confidence interval cut-off of 90% was chosen based on previous molecular studies. The sequence variation in ITS1 among thirty samples of *Croton stellatopilosus* collected from different parts of Thailand shows that these can be divided into three groups based on the occurrence of indels [52].

**Table 5:** Variable sites found between sequence pairs of two species (total length 320 bp)

Species	PER	CAS	CAU	CRA	HUT	KON
PER						
CAS	15					
CAU	15	20				
CRA	16	21	6			
HUT	4	18	18	19		
KON	15	16	14	16	18	

PER = *Croton persimilis*, CAS = *C*. *cascarilloides*, CAU = *C*. *caudatus*, CRA = *C*. *crassifolius*, HUT = *C*. *hutchinsonianus*, KON = *C*. *kongensis*

**Fig 5:**
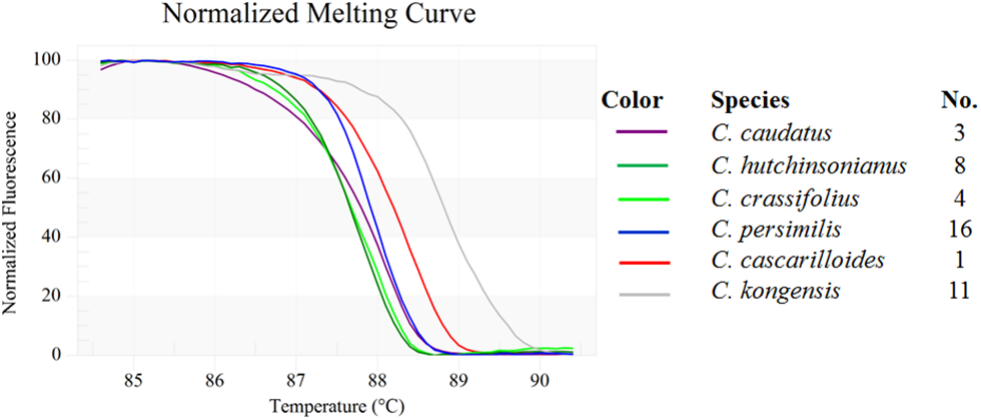
Melting profiles of ITS1 amplicons from six *Croton* species generated from HRM analysis using ITS1 primers.

**Fig 6:**
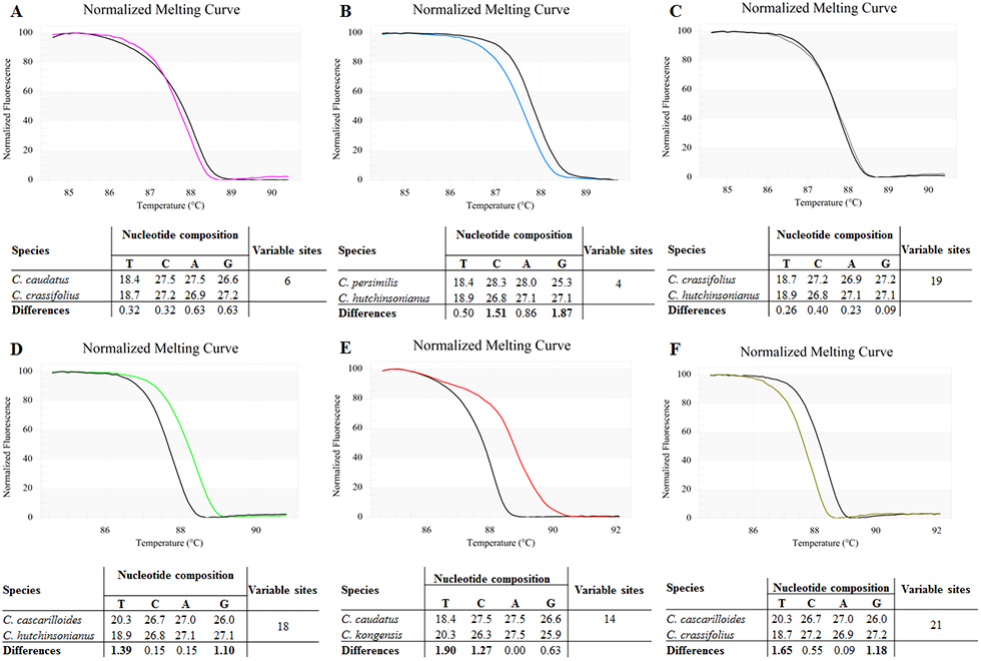
Normalized curves of pairwise comparisons between *Croton* ITS1 sequences, with a pairwise comparison of nucleotide composition and number of variable sites. **6A)** *C*. *caudatus* and *C*. *crassifolius*; **6B)** *C*. *hutchinsonianus* and *C*. *persimils*; **6C)** *C*. *crassifolius* and *C*. *hutchinsonianus*; **6D)** *C*. *cascarilloides* and *C*. *hutchinsonianus*; **6E)** *C*. *caudatus* and *C*. *kongensis*; **6F)** *C*. *cascarilloides* and *C*. *crassifolius*.

The melting profiles of the six *Croton* species (Fig 5) were compared to the pairwise sequence analyses (Table 5). As expected from the sequence analyses, melting curves of *C*. *caudatus* and *C*. *crassifolius* were nearly the same (6 variable sites in 320 bp fragment) (Fig 6A). The melting curves of *C*. *hutchinsonianus* and *C*. *persimilis* (4 variable sites) were also expected to be similar to each other but the result showed that they are not (Fig 6B). The most surprising result emerging from the HRM analysis is that the melting curves of *C*. *crassifolius* and *C*. *hutchinsonianus* are nearly identical to each other (Fig 6C), despite the presence of 19 variable sites among the two species’ ITS1 sequences. Comparison of the base composition of the amplicons for each of the nucleotides (Fig 6A–6F) yielded a possible explanation for these unexpected results. A small number of nucleotide variations were found between *C*. *hutchinsonianus* and *C*. *persimilis* (4 variable sites; Fig 6B), but differences in their base composition are as high as other pairs with different melting curves, e.g. *C*. *cascarilloides* and *C*. *hutchinsonianus* (18 variable sites; Fig 6D), *C*. *caudatus* and *C*. *kongensis* (14 variable sites; Fig 6E), and *C*. *cascarilloides* and *C*. *crassifolius* (21 variable sites; Fig 6F). It can thus be suggested that in the *C*. *crassifolius* and *C*. *hutchinsonianus* pair, where a high number of variable sites was found between the two sequences, low differences in the overall inter-sequence base composition resulted in similar melting curve shapes. This combination of findings provides some support for the conceptual premise that it is not only the nucleotide variation but also the nucleotide composition of each sample that contributes to the melting curve shape. Our results seem to be consistent with other research which found that DNA duplex stability can be determined by both sequence and base composition as in the nearest-neighbor (NN) model [53–55]. The NN model for nucleic acids assumes that the stability of a given base pair depends on the identity and orientation of neighboring base pairs. The NN model has long been engaged in several works on DNA melting analysis (e.g. [56–58]), even before the introduction of HRM technology. Several computer programs such as OligoCalc [59], uMELT [60] and MELTING [61] that can be used to predict melting temperatures and/or generate melting curves for DNA duplexes of interest are built on the NN model, and it is one of the most frequent approaches used for predicting melting temperature.

Although the ITS1 was found to be optimal primer pair in this study, it is likely that other markers and/or combinations thereof might perform better in other plant groups as the results shown in this study are limited to the examined genus (*Croton*).

## Conclusions

Bar-HRM has proven to be a cost-effective and reliable method for the identification of the twelve *Croton* species tested in this study. The hybrid method of DNA barcoding and High Resolution Melting is dependable, fast, and sensitive enough to distinguish between species. The fragment amplified using the ITS1 primer set had the highest single marker species discrimination rate out of the seven regions coming from the tested plastid and nuclear primer pairs, and it is likely that other ITS1 based primer sets will perform well in other plant groups. The Bar-HRM primer sets developed in this study are not only useful for identification of *Croton* plant vouchers, but can also be used for species discrimination, authentication, and detection of adulteration in samples lacking diagnostic morphological characters, such as herbal products.

## Supporting Information

S1 Table *Croton* sequences of *matK*, *rbcL*, *trnL* and ITS were retrieved from GenBank (NCBI) for each of the species with accession number. (DOCX)

## References

[1] Webster GL. A provisional synopsis of the sections of the genus *Croton* (Euphorbiaceae). Taxon. 1993; 793–823.

[2] Govaerts R, Frodin DG, Radcliffe-Smith A, Carter S. World checklist and bibliography of Euphorbiaceae (with Pandaceae). 2000; Available: http://agris.fao.org/agris-search/search.do?recordID=US201300050879

[3] Chayamarit K, van Welzen PC. Flora of Thailand, Volume 8, Part 1: Euphorbiaceae (Genera A-F). Bangkok, Thailand: Bangkok Forest Herbarium; 2005.

[4] Berry PE, Hipp AL, Wurdack KJ, Van Ee B, Riina R. Molecular phylogenetics of the giant genus *Croton* and tribe Crotoneae (Euphorbiaceae sensu stricto) using ITS and trnL-trnF DNA sequence data. Am J Bot. 2005;92: 1520–1534. 10.3732/ajb.92.9.1520 21646170

[5] Thongtan J, Kittakoop P, Ruangrungsi N, Saenboonrueng J, Thebtaranonth Y. New Antimycobacterial and Antimalarial 8, 9-Secokaurane Diterpenes from *Croton kongensis* . J Nat Prod. 2003;66: 868–870. 12828479 10.1021/np030067a

[6] Dey S, Mukherjee D, Chakraborty S, Mallick S, Dutta A, Ghosh J, et al Protective effect of *Croton caudatus* Geisel leaf extract against experimental visceral leishmaniasis induces proinflammatory cytokines in vitro and in vivo. Exp Parasitol. 2015;151: 84–95. 10.1016/j.exppara.2015.01.012 25655407

[7] Sun L, Meng Z, Li Z, Yang B, Wang Z, Ding G, et al Two new natural products from *Croton kongensis* Gagnep. Nat Prod Res. 2014;28: 563–567. 10.1080/14786419.2013.867856 24684202

[8] Ahmed B, Alam T, Varshney M, Khan SA. Hepatoprotective activity of two plants belonging to the Apiaceae and the Euphorbiaceae family. J Ethnopharmacol. 2002;79: 313–316. 11849834 10.1016/s0378-8741(01)00392-0

[9] El-Mekkawy S, Meselhy MR, Nakamura N, Hattori M, Kawahata T, Otake T. Anti-HIV-1 phorbol esters from the seeds of *Croton tiglium* . Phytochemistry. 2000;53: 457–464. 10731023 10.1016/s0031-9422(99)00556-7

[10] Minh PTH, Ngoc PH, Taylor WC, Cuong NM. A new ent-kaurane diterpenoid from *Croton tonkinensis* leaves. Fitoterapia. 2004;75: 552–556. 15351108 10.1016/j.fitote.2004.04.009

[11] Rates SMK. Plants as source of drugs. Toxicon. 2001;39: 603–613. 11072038 10.1016/s0041-0101(00)00154-9

[12] Kool A, de Boer HJ, Krüger Å, Rydberg A, Abbad A, Björk L, et al Molecular identification of commercialized medicinal plants in Southern Morocco. PLOS ONE. 2012;7: e39459 10.1371/journal.pone.0039459 22761800 PMC3384669

[13] Veldman S, Otieno J, Gravendeel B, Andel T van, de Boer HJ. Conservation of Endangered Wild Harvested Medicinal Plants: Use of DNA Barcoding. Gurib-Fakim A, editor. Nov Plant Bioresour Appl Food Med Cosmet. 2014; 81–88.

[14] Skalli S, Alaoui I, Pineau A, Zaid A, Soulaymani R. L’intoxication par le chardon à glu (*Atractylis gummifera* L.): à propos d’un cas clinique. Bull Soc Pathol Exot. 2002;95: 284–286. 12596380

[15] Ize-Ludlow D, Ragone S, Bruck IS, Bernstein JN, Duchowny M, Pena BMG. Neurotoxicities in infants seen with the consumption of star anise tea. Pediatrics. 2004;114: e653 10.1542/peds.2004-0058 15492355

[16] Ouarghidi A, Powell B, Martin GJ, de Boer HJ, Abbad A. Species substitution in medicinal roots and possible implications for toxicity in Morocco. Econ Bot. 2012;66: 370–382.

[17] Chen S, Yao H, Han J, Liu C, Song J, Shi L, et al Validation of the ITS2 region as a novel DNA barcode for identifying medicinal plant species. PLoS ONE. 2010;5: 1–8. 10.1371/journal.pone.0008613 PMC2799520 20062805

[18] Coghlan M, Haile J, Houston J, Murray D, White N, Moolhuijzen P, et al Deep sequencing of plant and animal DNA contained within traditional chinese medicines reveals legality issues and health safety concerns. PLoS Genet. 2012;8: e1002657 10.1371/journal.pgen.1002657 22511890 PMC3325194

[19] De Boer HJ, Ouarghidi A, Martin G, Abbad A, Kool A. DNA Barcoding Reveals Limited Accuracy of Identifications Based on Folk Taxonomy. PLOS ONE. 2014;9: e84291 10.1371/journal.pone.0084291 24416210 PMC3885563

[20] Newmaster SG, Grguric M, Shanmughanandhan D, Ramalingam S, Ragupathy S. DNA barcoding detects contamination and substitution in North American herbal products. BMC Med. 2013;11: 222 10.1186/1741-7015-11-222 24120035 PMC3851815

[21] Li M, Cao H, But PP-H, Shaw P-C. Identification of herbal medicinal materials using DNA barcodes. J Syst Evol. 2011;49: 271–283.

[22] Techen N, Parveen I, Pan Z, Khan IA. DNA barcoding of medicinal plant material for identification. Curr Opin Biotechnol. 2014;25: 103–110. 10.1016/j.copbio.2013.09.010 24484887

[23] De Boer HJ, Ichim MC, Newmaster SG. DNA Barcoding and Pharmacovigilance of Herbal Medicines. Drug Saf. [In Press]; 10.1007/s40264-015-0306-8 26076652

[24] Särkinen T, Staats M, Richardson JE, Cowan RS, Bakker FT. How to Open the treasure chest? Optimising DNA extraction from herbarium specimens. PLOS ONE. 2012;7: e43808 10.1371/journal.pone.0043808 22952770 PMC3429509

[25] Piredda R, Simeone MC, Attimonelli M, Bellarosa R, Schirone B. Prospects of barcoding the Italian wild dendroflora: oaks reveal severe limitations to tracking species identity. Mol Ecol Resour. 2011;11: 72–83. 10.1111/j.1755-0998.2010.02900.x 21429102

[26] Sass C, Little DP, Stevenson DW, Specht CD. DNA barcoding in the cycadales: testing the potential of proposed barcoding markers for species identification of cycads. PLOS ONE. 2007;2: e1154 10.1371/journal.pone.0001154 17987130 PMC2063462

[27] Stoeckle MY, Gamble CC, Kirpekar R, Young G, Ahmed S, Little DP. Commercial teas highlight plant DNA barcode identification successes and obstacles. Sci Rep. 2011;1: 1–7. 22355561 10.1038/srep00042 PMC3216529

[28] Fazekas AJ, Kesanakurti PR, Burgess KS, Percy DM, Graham SW, Barrett SCH, et al Are plant species inherently harder to discriminate than animal species using DNA barcoding markers? Mol Ecol Resour. 2009;9: 130–139. 10.1111/j.1755-0998.2009.02652.x 21564972

[29] Osathanunkul M, Madesis P, de Boer HJ. Bar-HRM for authentication of plant-based medicines: evaluation of three medicinal products derived from Acanthaceae species. PLoS ONE. 2015; 10 (5): e0128476 10.1371/journal.pone.0128476 26011474 PMC4444109

[30] Reja V, Kwok A, Stone G, Yang L, Missel A, Menzel C, et al ScreenClust: Advanced statistical software for supervised and unsupervised high resolution melting (HRM) analysis. Methods. 2010;50: S10–S14. 10.1016/j.ymeth.2010.02.006 20146938

[31] Reed GH, Wittwer CT. Sensitivity and specificity of single-nucleotide polymorphism scanning by high-resolution melting analysis. Clin Chem. 2004;50: 1748–1754. 15308590 10.1373/clinchem.2003.029751

[32] Ririe KM, Rasmussen RP, Wittwer CT. Product differentiation by analysis of DNA melting curves during the polymerase chain reaction. Anal Biochem. 1997;245: 154–160. 9056205 10.1006/abio.1996.9916

[33] Wittwer CT, Reed GH, Gundry CN, Vandersteen JG, Pryor RJ. High-resolution genotyping by amplicon melting analysis using LCGreen. Clin Chem. 2003;49: 853–860. 12765979 10.1373/49.6.853

[34] Madesis P, Ganopoulos I, Sakaridis I, Argiriou A, Tsaftaris A. Advances of DNA-based methods for tracing the botanical origin of food products. Food Res Int. 2014;60: 163–172.

[35] Ganopoulos I, Madesis P, Darzentas N, Argiriou A, Tsaftaris A. Barcode High Resolution Melting (Bar-HRM) analysis for detection and quantification of PDO “Fava Santorinis” (*Lathyrus clymenum*) adulterants. Food Chem. 2012;133: 505–512. 10.1016/j.foodchem.2012.01.015 25683426

[36] Ganopoulos I, Bazakos C, Madesis P, Kalaitzis P, Tsaftaris A. Barcode DNA high-resolution melting (Bar-HRM) analysis as a novel close-tubed and accurate tool for olive oil forensic use. J Sci Food Agric. 2013;93: 2281–2286. 10.1002/jsfa.6040 23400707

[37] Ganopoulos I, Xanthopoulou A, Mastrogianni A, Drouzas A, Kalivas A, Bletsos F, et al High Resolution Melting (HRM) analysis in eggplant (*Solanum melongena* L.): A tool for microsatellite genotyping and molecular characterization of a Greek Genebank collection. Biochem Syst Ecol. 2015;58: 64–71.

[38] Kalivas A, Ganopoulos I, Xanthopoulou A, Chatzopoulou P, Tsaftaris A, Madesis P. DNA barcode ITS2 coupled with high resolution melting (HRM) analysis for taxonomic identification of *Sideritis* species growing in Greece. Mol Biol Rep. 2014;41: 5147–5155. 10.1007/s11033-014-3381-5 24802796

[39] Schmiderer C, Mader E, Novak J. DNA-based identification of *Helleborus* niger by high-resolution melting analysis. Planta Med. 2010;76: 1934–1937. 10.1055/s-0030-1249908 20455201

[40] Ganopoulos I, Aravanopoulos F, Madesis P, Pasentsis K, Bosmali I, Ouzounis C, et al Taxonomic identification of Mediterranean pines and their hybrids based on the high resolution melting (HRM) and trnL approaches: from cytoplasmic inheritance to timber tracing. PLOS ONE. 2013;8: e60945 10.1371/journal.pone.0060945 23577179 PMC3618329

[41] Madesis P, Ganopoulos I, Bosmali I, Tsaftaris A. Barcode High Resolution Melting analysis for forensic uses in nuts: A case study on allergenic hazelnuts (*Corylus avellana*). Food Res Int. 2013;50: 351–360.

[42] Madesis P, Ganopoulos I, Anagnostis A, Tsaftaris A. The application of Bar-HRM (Barcode DNA-High Resolution Melting) analysis for authenticity testing and quantitative detection of bean crops (Leguminosae) without prior DNA purification. Food Control. 2012;25: 576–582.

[43] Bosmali I, Ganopoulos I, Madesis P, Tsaftaris A. Microsatellite and DNA-barcode regions typing combined with High Resolution Melting (HRM) analysis for food forensic uses: A case study on lentils (*Lens culinaris*). Food Res Int. 2012;46: 141–147.

[44] Ganopoulos I, Madesis P, Tsaftaris A. Universal ITS2 barcoding DNA region coupled with high-resolution melting (HRM) analysis for seed authentication and adulteration testing in leguminous forage and pasture species. Plant Mol Biol Report. 2012;30: 1322–1328.

[45] Tamura K, Peterson D, Peterson N, Stecher G, Nei M, Kumar S. MEGA5: molecular evolutionary genetics analysis using maximum likelihood, evolutionary distance, and maximum parsimony methods. Mol Biol Evol. 2011;28: 2731–2739. 10.1093/molbev/msr121 21546353 PMC3203626

[46] CBOL Plant Working Group. A DNA barcode for land plants. Proc Natl Acad Sci. 2009;106: 12794–12797. 10.1073/pnas.0905845106 19666622 PMC2722355

[47] Hollingsworth PM. Refining the DNA barcode for land plants. Proc Natl Acad Sci U S A. 2011;108: 19451–19452. 10.1073/pnas.1116812108 22109553 PMC3241790

[48] Fazekas AJ, Burgess KS, Kesanakurti PR, Graham SW, Newmaster SG, Husband BC, et al Multiple multilocus DNA barcodes from the plastid genome discriminate plant species equally well. PLOS ONE. 2008;3: e2802 10.1371/journal.pone.0002802 18665273 PMC2475660

[49] Kress WJ, Wurdack KJ, Zimmer EA, Weigt LA, Janzen DH. Use of DNA barcodes to identify flowering plants. Proc Natl Acad Sci. 2005;102: 8369–8374. 15928076 10.1073/pnas.0503123102 PMC1142120

[50] Li DZ, Gao LM, Li HT, Wang H, Ge XJ, Liu JQ, et al Comparative analysis of a large dataset indicates that internal transcribed spacer (ITS) should be incorporated into the core barcode for seed plants. Proc Natl Acad Sci. 2011;108: 19641–19646. 10.1073/pnas.1104551108 22100737 PMC3241788

[51] Gao T, Yao H, Song J, Liu C, Zhu Y, Ma X, et al Identification of medicinal plants in the family Fabaceae using a potential DNA barcode ITS2. J Ethnopharmacol. 2010;130: 116–121. 10.1016/j.jep.2010.04.026 20435122

[52] Rinthong P, Zhu S, Komatsu K, Chanama S, De-Eknamkul W. Genetic variation of *Croton stellatopilosus* Ohba based on non-coding DNA sequences of ITS, trnK and trnL-F regions. J Nat Med. 2011;65: 641–645. 10.1007/s11418-011-0536-8 21499847

[53] Sugimoto N, Katoh M, Nakano S, Ohmichi T, Sasaki M. RNA/DNA hybrid duplexes with identical nearest-neighbor base-pairs have identical stability. FEBS Lett. 1994;354: 74–78. 7525350 10.1016/0014-5793(94)01098-6

[54] SantaLucia J, Allawi HT, Seneviratne PA. Improved nearest-neighbor parameters for predicting DNA duplex stability. Biochemistry (Mosc). 1996;35: 3555–3562. 10.1021/bi951907q 8639506

[55] Turner DH, Mathews DH. NNDB: the nearest neighbor parameter database for predicting stability of nucleic acid secondary structure. Nucleic Acids Res. 2009; gkp892. 10.1093/nar/gkp892 PMC2808915 19880381

[56] DeVoe H, Tinoco I. The stability of helical polynucleotides: base contributions. J Mol Biol. 1962;4: 500–517. 13885894 10.1016/s0022-2836(62)80105-3

[57] Gray DM, Tinoco I. A new approach to the study of sequence-dependent properties of polynucleotides. Biopolymers. 1970;9: 223–244.

[58] Gotoh O, Tagashira Y. Stabilities of nearest-neighbor doublets in double-helical DNA determined by fitting calculated melting profiles to observed profiles. Biopolymers. 1981;20: 1033–1042.

[59] Kibbe WA. OligoCalc: an online oligonucleotide properties calculator. Nucleic Acids Res. 2007;35: W43–W46. 17452344 10.1093/nar/gkm234 PMC1933198

[60] Dwight Z, Palais R, Wittwer CT. uMELT: prediction of high-resolution melting curves and dynamic melting profiles of PCR products in a rich web application. Bioinformatics. 2011;27: 1019–1020. 10.1093/bioinformatics/btr065 21300699

[61] Dumousseau M, Rodriguez N, Juty N, Novère NL. MELTING, a flexible platform to predict the melting temperatures of nucleic acids. BMC Bioinformatics. 2012;13: 101 10.1186/1471-2105-13-101 22591039 PMC3733425

