# Supplementary material for: Refining DNA Barcoding Coupled High Resolution Melting for Discrimination of 12 Closely Related Croton Species
Source: PLoS One. 2015 Sep 25;10(9):e0138888. doi: 10.1371/journal.pone.0138888 (PMC4583236; doi:10.1371/journal.pone.0138888)
Supplement: S1 Table — (DOCX) [file pone.0138888.s001.docx]

**S1 Table.** *Croton* sequences of *matK, rbcL, trnL* and ITS were retrieved from GenBank (NCBI) for each of the species with accession number.

| **scienctific names** | **Accession number (NCBI)** | | | | **scienctific names** | **Accession number (NCBI)** | | | |
| --- | --- | --- | --- | --- | --- | --- | --- | --- | --- |
|  | *matK* | *rbcL* | *trnL* | ITS |  | *matK* | *rbcL* | *trnL* | ITS |
| *Croton abutiloides* | - | - | - | EU586903 | *Croton campanulatus* | - | - | HM044771 | HM044790 |
| *Croton acapulcensis* | - | - | - | EU477862 | *Croton capitatus* | - | - | EU478167 | EU478105 |
| *Croton adenophyllus* | - | - | EU497700 | - | *Croton caracasanus* | - | EF405834 | - | DQ227525 |
| *Croton adspersus* | - | - | EU478129 | - | *Croton cascarilla* | - | - | - | EU477933 |
| *Croton aequatoris* | - | - | - | EU586904 | *Croton cascarilloides* | - | - | AB375097 | AY971191 |
| *Croton alamosanus* | - | - | EU478123 | - | *Croton catamarcensis* | - | - | - | HM071949 |
| *Croton alchorneicarpus* | - | - | HM044769 | - | *Croton caudatus* | - | - | - | AY971192 |
| *Croton ameliae* | - | - | FJ614771 | - | *Croton ceanothifolius* | - | - | - | HM071950 |
| *Croton andinus* | - | - | FJ614805 | - | *Croton celtidifolius* | - | - | EU586975 | EU586920 |
| *Croton antisyphiliticus* | - | - | AY971180 | - | *Croton cerinus* | - | - | - | FJ614705 |
| *Croton arboreus* | - | - | EU497701 | - | *Croton chamelensis* | - | - | - | EU478060 |
| *Croton argenteus* | - | - | EU497702 | - | *Croton chichenensis* | - | - | EU478130 | EU477901 |
| *Croton argentinus* | - | - | HM564210 | HM071943 | *Croton chilensis* | - | - | EU586959 | EU586905 |
| *Croton argyranthemus* | - | - | HM564211 | EU478009 | *Croton chimboracensis* | - | - | - | AY971204 |
| *Croton argyratus* | - | - | AB375095 | - | *Croton chocoanus* | - | - | - | EU586941 |
| *Croton argyrophyllus* | - | - | HM564213 | - | *Croton ciliatoglandulifer* | - | - | EU478146 | EU477997 |
| *Croton astroites* | - | KJ082246 | - | EU586901 | *Croton columnaris* | - | AB925360 | AB375099 | - |
| *Croton ater* | - | - | - | EU586952 | *Croton conduplicatus* | - | - | - | EU477957 |
| *Croton axillaris* | - | - | - | EU477865 | *Croton coriaceus* | - | - | - | EU586921 |
| *Croton beetlei* | - | - | EF408108 | - | *Croton coriifolius* | - | EF405835 | - | AY971199 |
| *Croton betulinus* | - | KJ082247 | - | FJ604248 | *Croton coryi* | - | - | EU478152 | EU478012 |
| *Croton bigbendensis* | - | - | - | EU478011 | *Croton corylifolius* | - | - | EF408094 | - |
| *Croton billbergianus* | JQ587440 | JQ591428 | - | - | *Croton corinthius* | - | - | EF405835 | EF408110 |
| *Croton bispinosus* | - | - | EU497704 | - | *Croton coryi* | - | - | - | EU478152 |
| *Croton bixoides* | - | - | - | AY971184 | *Croton craspedotrichus* | - | - | - | DQ227532 |
| *Croton boiteaui* | - | - | HM071966 | HM071944 | *Croton crassifolius* | - | - | - | AY971200 |
| *Croton bojerianus* | - | - | HM071967 | HM071945 | *Croton crocodilorum* | - | - | - | AY971201 |
| *Croton bonplandianus* | - | KF425767 | - | AY971185 | *Croton culiacanensis* | - | - | - | EU477866 |
| *Croton bracteatus* | - | - | - | AY971186 | *Croton cuneatus* | - | - | - | EU478005 |
| *Croton bredemeyeri* | - | - | - | AY971187 | *Croton cupreatus* | - | - | HM564214 | EU586919 |
| *Croton brevipes* | - | - | - | EU478058 | *Croton cupulifer* | - | - | - | EU478063 |
| *Croton brittonianus* | - | - | EU497706 | - | *Croton curiosus* | - | - | - | EU586906 |
| *Croton burchellii* | - | - | - | HM071947 | *Croton cuyabensis* | - | - | - | HM071951 |
| *Croton caboensis* | - | - | - | EU477897 | *Croton daphniphyllum* | - | EF405836 | - | DQ227531 |
| *Croton cajucara* | - | - | EU586968 | EU586913 | *Croton decalobus* | - | - | - | EU477869 |
| *Croton californicus* | - | - | - | AY971189 | *Croton decalvatus* | - | - | AB375110 | - |

**S1 Table.** (Continue) *Croton* sequences of *matK, rbcL, trnL* and ITS were retrieved from GenBank (NCBI) for each of the species with accession number.

| **scienctific names** | **Accession number (NCBI)** | | | | **scienctific names** | **Accession number (NCBI)** | | | |
| --- | --- | --- | --- | --- | --- | --- | --- | --- | --- |
|  | *matK* | *rbcL* | *trnL* | ITS |  | *matK* | *rbcL* | *trnL* | ITS |
| *Croton dichrous* | - | - | - | HM071952 | *Croton gynopetalus* | - | - | - | EU478069 |
| *Croton dioicus* | - | - | - | EU478014 | *Croton helicoideus* | - | - | EU586956 | EU586902 |
| *Croton discolor* | - | KJ082248 | - | - | *Croton heliotropiifolius* | - | - | AY794695 | - |
| *Croton disjunctus* | - | - | - | EU477903 | *Croton hemiargyreus* | - | - | HM044774 | HM044793 |
| *Croton domatifer* | - | - | GU296103 | GU296102 | *Croton heteranthus* | - | - | - | AY971216 |
| *Croton draco* | - | EF405840 | EF408114 | EU478006 | *Croton heterocalyx* | - | - | HM044775 | - |
| *Croton draconoides* | - | - | - | EU586978 | *Croton heterochrous* | - | - | - | EU477920 |
| *Croton echinocarpus* | - | - | EU586979 | EU586922 | *Croton hibiscifolius* | - | - | - | EU586925 |
| *Croton echioides* | - | - | EU586967 | EU586907 | *Croton hircinus* | - | - | EU478127 | EU477889 |
| *Croton ehrenbergii* | - | - | - | EU477904 | *Croton hirtus* | - | - | EU478160 | EU478071 |
| *Croton eichleri* | - | - | EU587001 | EU586949 | *Croton hoffmannii* | - | EF405837 | EF408111 | - |
| *Croton ekmanii* |  | - | EF405860 | - | *Croton huberi* | - | - | - | AY971217 |
| *Croton elegans* | - | - | - | AY971207 | *Croton humilis* | - | - | HM564218 | EU477925 |
| *Croton elliottii* | - | - | - | EU478107 | *Croton hutchinsonianus* | - | - | - | AY971218 |
| *Croton emporiorum* | - | - | - | EU586908 | *Croton hypoleucus* | - | - | - | EU477989 |
| *Croton eremophilus* | - | - | HM564216 | - | *Croton icche* | - | - | - | EU478030 |
| *Croton erythroxyloides* | - | - | EU586992 | EU586938 | *Croton impressus* | - | KJ082254 | EF408113 | - |
| *Croton fantzianus* | - | - | - | EU478032 | *Croton insularis* | AB233773 | KF496848 | - | AY971220 |
| *Croton flavens* | KJ012553 | - | EU478134 | EU477907 | *Croton itzaeus* | - | - | EU478161 | EU478072 |
| *Croton flaviglandulosus* | - | - | - | EU478064 | *Croton jacobinensis* | - | - | HM044776 | HM044795 |
| *Croton floccosus* | - | - | EU586980 | EU586923 | *Croton jimenezii* | - | EF405841 | EF408115 | EU478007 |
| *Croton fragrans* | - | - | HM044772 | EU478000 | *Croton jutiapensis* | JQ587443 | JQ591441 | - | EU478076 |
| *Croton fragrantulus* | - | - | - | HM044792 | *Croton kerrii* | - | - | - | AY971221 |
| *Croton francoanus* | - | - | - | EU477915 | *Croton klotzschianus* | - | - | - | AY971222 |
| *Croton fruticulosus* | - | - | EU478136 | EU477918 | *Croton kongensis* | GQ434077 | - | - | AY971223 |
| *Croton fuscescens* | - | - | HM564217 | - | *Croton kongkandanus* | - | - | AB375111 | - |
| *Croton glabellus* | KJ012554 | KJ082252 | - | - | *Croton laceratoglandulosus* | - | - | - | DQ836744 |
| *Croton glandulosepalus* | - | - | EU478126 | EU477888 | *Croton lachnocarpus* | HQ415239 | HQ415051 | - | AY971224 |
| *Croton glandulosus* | - | - | EU478159 | EU478057 | *Croton lagoensis* | - | - | - | EU586926 |
| *Croton gossypiifolius* | - | - | EU586981 | EU586924 | *Croton lasiopetaloides* | - | - | - | EU477930 |
| *Croton goudotii* | - | - | - | EU586946 | *Croton laureltyanus* | - | - | - | HM071953 |
| *Croton gracilipes* | - | - | EU586962 | EU586909 | *Croton laurinus* | - | EF405842 | - | - |
| *Croton gratissimus* | JX517905 | JX572483 | AY794696 | AY971214 | *Croton lechleri* | - | - | EU586983 | EU586927 |
| *Croton grisebachianus* | - | - | EU497714 | - | *Croton leonis* | - | - | EF408140 | - |
| *Croton guatemalensis* | - | - | - | EU478036 | *Croton lepidotus* | - | - | EU497718 | - |
| *Croton guianensis* | - | - | - | AY971215 | *Croton leptostachyus* | - | - | - | AY971226 |

**S1 Table.** (Continue) *Croton* sequences of *matK, rbcL, trnL* and ITS were retrieved from GenBank (NCBI) for each of the species with accession number.

| **scienctific names** | **Accession number (NCBI)** | | | | **scienctific names** | **Accession number (NCBI)** | | | |
| --- | --- | --- | --- | --- | --- | --- | --- | --- | --- |
|  | *matK* | *rbcL* | *trnL* | ITS |  | *matK* | *rbcL* | *trnL* | ITS |
| *Croton leucophyllus* | - | - | - | EU478109 | *Croton mongue* | - | - | - | EU586947 |
| *Croton liebmannii* | - | - | - | EU478077 | *Croton montevidensis* | - | - | - | AY971235 |
| *Croton limnocharis* | - | - | - | EU477931 | *Croton morifolius* | JQ587446 | JQ591444 | - | - |
| *Croton lindheimeri* | - | - | FJ614799 | EU478106 | *Croton myricifolius* | - | - | HM564226 | - |
| *Croton lindheimerianus* | - | - | FJ614788 | EU478111 | *Croton neomexicanus* | - | - | - | EU478016 |
| *Croton linearifolius* | - | - | HM071971 | HM071954 | *Croton nephrophyllus* | - | - | - | DQ227534 |
| *Croton lobatus* | - | - | AY794689 | - | *Croton niveus* | JQ587447 | EF405847 | EF408119 | EU478046 |
| *Croton louvelii* | - | - | HM564221 | - | *Croton nobilis* | - | - | HM044778 | HM044797 |
| *Croton lucidus* | - | AY794909 | AY794701 | - | *Croton noronhae* | - | - | EF408120 | - |
| *Croton luetzelburgii* | - | - | HM564222 | - | *Croton nubigenus* | - | EF405849 | EF408121 | EF421716 |
| *Croton lundellii* | - | EF405844 | EF408097 | - | *Croton nummulariifolius* | - | - | HM071973 | HM071956 |
| *Croton macrobothrys* | - | - | EU586984 | EU586928 | *Croton oerstedianus* | - | - | EF408106 | EF421717 |
| *Croton macrodontus* | - | - | - | EU478078 | *Croton olivaceus* | - | EF405830 | - | - |
| *Croton magdalenae* | - | - | - | EU477937 | *Croton oreades* | - | - | - | EU586948 |
| *Croton magdalenensis* | - | - | - | EU586929 | *Croton organensis* | - | - | EU586969 | EU586914 |
| *Croton maestrensis* | - | EF405857 | EF408127 | - | *Croton orinocensis* | - | - | HM044779 | HM044799 |
| *Croton malambo* | - | - | - | AY971228 | *Croton ortholobus* | - | - | EU478163 | EU478061 |
| *Croton malvaviscifolius* | - | - | EU478162 | EU478080 | *Croton ovalifolius* | - | - | - | AY971238 |
| *Croton manampetsae* | - | - | HM044777 | HM044796 | *Croton pachypodus* | - | - | EF408128 | - |
| *Croton martinianus* | - | - | - | AY971229 | *Croton pachysepalus* | - | - | - | HM071957 |
| *Croton masonii* | - | - | - | EU477871 | *Croton palanostigma* | - | - | EU586997 | EU586943 |
| *Croton matourensis* | - | - | - | EU478097 | *Croton pallidulus* | - | - | EU586993 | EU586939 |
| *Croton mayarum* | - | - | - | EU478038 | *Croton paludosos* | - | - | - | DQ787388 |
| *Croton medusae* | - | - | EU586988 | EU586933 | *Croton parksii* | - | - | - | EU478019 |
| *Croton megalobotrys* | JX517792 | EU213464 | - | - | *Croton payaquensis* | - | - | - | EU477951 |
| *Croton megalodendron* | - | - | EU586996 | EU586942 | *Croton pedicellatus* | - | - | FJ614804 | EU478121 |
| *Croton megistocarpus* | - | JQ591443 | EF408129 | EU586953 | *Croton peltoideus* | - | - | HM071975 | HM071958 |
| *Croton menyharthii* | KF147385 | JF265377 | - | - | *Croton pendens* | - | - | - | EU477872 |
| *Croton mexicanus* | - | - | EF408107 | - | *Croton peraeruginosus* | - | - | EU478141 | EU477919 |
| *Croton miarensis* | - | - | HM564223 | - | *Croton peraeruginosus* | - | - | - | EU477953 |
| *Croton micans* | - | - | - | AY971232 | *Croton persimilis* | - | KF523366 | - | - |
| *Croton michaelii* | - | - | EU478139 | EU477940 | *Croton perspeciosus* | - | - | EU586986 | EU586931 |
| *Croton michauxii* | - | - | - | AY971233 | *Croton piluliferus* | - | - | EU586987 | EU586932 |
| *Croton microcarpus* | - | - | HM071972 | HM071955 | *Croton piptocalyx* | - | - | EF408148 | - |
| *Croton microtiglium* | - | - | - | AY971234 | *Croton poecilanthus* | HM446680 | EF405850 | EF408122 | - |
| *Croton monanthogynus* | - | - | EU478169 | EU478114 | *Croton poilanei* | - | AB925535 | AB375102 | AY971239 |

**S1 Table.** (Continue) *Croton* sequences of *matK, rbcL, trnL* and ITS were retrieved from GenBank (NCBI) for each of the species with accession number.

| **scienctific names** | **Accession number (NCBI)** | | | | **scienctific names** | **Accession number (NCBI)** | | | |
| --- | --- | --- | --- | --- | --- | --- | --- | --- | --- |
|  | *matK* | *rbcL* | *trnL* | ITS |  | *matK* | *rbcL* | *trnL* | ITS |
| *Croton polyandrus* | - | - | HM564229 | - | *Croton setigerus* | - | AY794697 | - | - |
| *Croton populifolius* | - | - | EU497724 | - | *Croton skutchii* | - | - | - | EU478100 |
| *Croton pottsii* | - | - | - | EU478119 | *Croton smithianus* | - | - | - | EU478101 |
| *Croton priscus* | - | - | - | EU586950 | *Croton socotranus* | - | - | - | AY971250 |
| *Croton pseudofragrans* | - | - | HM044780 | HM044800 | *Croton soliman* | - | - | - | EU477965 |
| *Croton pseudoniveus* | - | - | - | EU478049 | *Croton sonorae* | - | - | EU478143 | EU477967 |
| *Croton pseudopulchellus* | EU214238 | EU213467 | - | - | *Croton soratensis* | - | - | HM071979 | HM071961 |
| *Croton pulcher* | - | - | EU478147 | EU477995 | *Croton sousae* | - | - | - | EU478054 |
| *Croton punctatus* | - | - | EF408123 | EU478022 | *Croton speciosus* | - | - | AY794699 | AY971251 |
| *Croton pungens* | - | - | - | AY971241 | *Croton sphaerogynus* | - | KF981219 | HM044784 | HM044805 |
| *Croton purdiei* | - | - | - | EU586934 | *Croton spruceanus* | - | - | HM044785 | HM044806 |
| *Croton pycnocephalus* | - | - | FJ614776 | FJ614708 | *Croton steenkampianus* | JX517563 | JX572487 | - | - |
| *Croton ramillatus* | - | - | - | EU478082 | *Croton stellatopilosus* | AB428645 | - | AB375106 | - |
| *Croton redolens* | - | - | - | EU586935 | *Croton stipulaceus* | - | - | - | EU477975 |
| *Croton reflexifolius* | - | - | - | EU478056 | *Croton suaveolens* | - | - | - | EU477977 |
| *Croton repens* | - | - | HM071976 | EU478089 | *Croton suberosus* | - | - | EU478144 | EU477973 |
| *Croton rimbachii* | - | - | EU586990 | EU586936 | *Croton subpannosus* | - | - | - | HM071962 |
| *Croton ripensis* | - | - | - | AY971243 | *Croton sutup* | - | - | EU478128 | EU477894 |
| *Croton roraimensis* | - | - | EF408149 | - | *Croton suyapensis* | - | - | - | EU477980 |
| *Croton rosmarinoides* | - | - | EU497725 | - | *Croton sylvaticus* | JF270732 | JX572488 | - | - |
| *Croton rottlerifolius* | - | - | HM044781 | HM044801 | *Croton tabascensis* | - | - | - | EU477875 |
| *Croton roxburghii* | - | - | AB375105 | AY971244 | *Croton tenuilobus* | - | - | FJ614803 | FJ614768 |
| *Croton ruizianus* | - | - | - | EU586910 | *Croton texensis* | - | - | - | EU478023 |
| *Croton sacaquinha* | - | - | HM044782 | HM044802 | *Croton thomasii* | - | - | EF408151 | EU586951 |
| *Croton saltensis* | - | - | - | EU586911 | *Croton tiglium* | - | GQ436320 | AB375107 | - |
| *Croton salutaris* | - | - | HM071977 | HM044804 | *Croton torreyanus* | - | - | - | EU477981 |
| *Croton sampatik* | - | - | EF408133 | - | *Croton trichotomus* | - | - | HM044787 | HM044808 |
| *Croton sanctilazari* | - | - | - | EU477964 | *Croton tricolor* | - | - | EF408125 | - |
| *Croton santaritensis* | - | - | - | EU478090 | *Croton trigonocarpus* | - | EF405861 | - | - |
| *Croton sapiifolius* | - | - | EF408150 | - | *Croton trinitatis* | - | - | - | EU478092 |
| *Croton sarcopetalus* | - | - | - | EU586912 | *Croton triqueter* | - | - | - | AY971256 |
| *Croton scaber* | - | - | - | EU477874 | *Croton troncosoi* | - | - | EU586994 | EU586940 |
| *Croton schiedeanus* | - | JQ591457 | - | EU478052 | *Croton urucurana* | - | - | EU586991 | - |
| *Croton sellowii* | - | - | HM564230 | - | *Croton varelae* | - | - | - | DQ227536 |
| *Croton serratifolius* | - | - | HM071978 | HM071960 | *Croton velutinus* | - | - | HM044786 | HM044807 |
| *Croton setiger* | - | - | - | AY971249 | *Croton vernicosa* | - | - | KC479246 | - |

**S1 Table.** (Continue) *Croton* sequences of *matK, rbcL, trnL* and ITS were retrieved from GenBank (NCBI) for each of the species with accession number.

| **scienctific names** | **Accession number (NCBI)** | | | |
| --- | --- | --- | --- | --- |
|  | *matK* | *rbcL* | *trnL* | ITS |
| *Croton verreauxii* | - | - | - | AY971257 |
| *Croton virletianus* | - | - | - | EU477982 |
| *Croton vitifolius* | - | - | - | EU477984 |
| *Croton wallichii* | - | - | AB375108 | - |
| *Croton warmingii* | - | - | EU586970 | EU586915 |
| *Croton watsonii* | - | - | FJ614769 | EU477882 |
| *Croton websteri* | - | - | - | EU477986 |
| *Croton wigginsii* | - | - | - | EU478024 |
| *Croton willdenowii* | - | - | EU478149 | EU478004 |
| *Croton wilsonii* | - | - | EU497726 | - |
| *Croton womersleyi* | - | JF738754 | - | - |
| *Croton xalapensis* | - | - | - | EU477988 |
| *Croton yavitensis* | - | - | EU586973 | EU586918 |
| *Croton yecorensis* | - | - | - | AY971258 |
| *Croton ynesae* | - | - | FJ614772 | - |
| *Croton yucatanensis* | JQ587450 | - | EF408126 | EU477880 |
| *Croton zambesicus* | - | - | - | AY971260 |
